# Soil Biodiversity Supports Agroecosystem Services Under Multiple Global Change Factors

**DOI:** 10.1111/gcb.71064

**Published:** 2026-08-20

**Authors:** Catalina Garzón‐Ladino, Guiyao Zhou, Daniel Revillini, Samuel Castejon, Yuanyuan Bao, Youzhi Feng, Manuel Delgado‐Baquerizo

**Affiliations:** ^1^ Laboratorio de Biodiversidad y Funcionamiento Ecosistémico. Instituto de Recursos Naturales y Agrobiología de Sevilla (IRNAS) Consejo Superior de Investigaciones Científicas (CSIC) Sevilla Spain; ^2^ Conservation of Natural Resources Department NEIKER—Basque Institute for Agricultural Research and Development, Basque Research and Technology Alliance (BRTA), Parque Científico y Tecnológico de Bizkaia Derio Spain; ^3^ State Key Laboratory for the Development and Utilization of Forest Food Resources Nanjing Forestry University Nanjing China

**Keywords:** agroecosystems, ecosystem multifunctionality, global change factors, microbial diversity, soil biodiversity

## Abstract

Cereals are the key staple foods supporting a growing population under global change. Soil biodiversity sustains ecosystem multifunctionality (EMF) that underpins food production and soil agroecosystem services. Yet, the capacity of soil biodiversity to support EMF in cereal fields under multiple global change factors (GCFs) remains virtually unknown. Here, we conducted a field mesocosm experiment involving wheat, four levels of soil biodiversity, and up to eight distinct GCFs (warming, drought, salinity, heavy metals, nitrogen addition, microplastics, pesticides, and alkalinization) to investigate the contributions of soil biodiversity in supporting EMF (e.g., crop productivity, nutrient cycling, plant symbionts, plant health and organic matter decomposition) under multiple GCFs. Our results reveal that soil biodiversity maintains positive effects on EMF under increasing levels of GCFs. Intermediate levels of multiple GCFs stimulated vegetative wheat biomass, likely due to compensatory responses, but often at the expense of other soil ecosystem functions such as organic matter decomposition or the prevalence of plant symbionts, highlighting the inherent trade‐offs under global change. These findings demonstrate that preserving soil biodiversity is essential to maintain agroecosystem functioning under multiple global change factors.

## Graphical abstract

Soil biodiversity is essential for maintaining healthy and productive agricultural systems under climate change. Using wheat fields exposed to up to eight global change factors, such as warming, drought, salinity, heavy metals, nitrogen addition, microplastics, pesticides, and alkalinization, we found that diverse soil communities play a critical role in sustaining multiple ecosystem functions, including crop growth, nutrient cycling, and soil health, highlighting that preserving soil biodiversity is essential to maintain agroecosystem functioning under multiple global change factors. Figure created with FigureLabs (figurelabs.ai) and assembled in Inkscape.

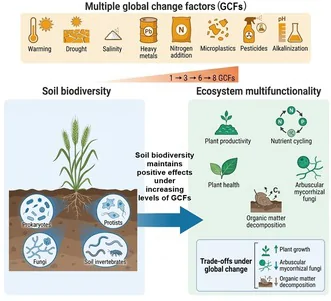

## 1 Introduction

Cereals are fundamental to maintain a growing human population under global change. Moreover, cereal fields provide multiple ecosystem functions simultaneously (multifunctionality; EMF), including soil biological activity, plant health and plant–soil symbiosis, to name a few. These ecosystem functions are critical for long‐term sustainability in agriculture. Unfortunately, cereal production is challenged by multiple global change factors (GCFs) (FSIN and GNAFC 2025) such as climate change, alkalinization, microplastics, salinification, over‐fertilization, pesticide accumulation, or soil contamination (Rillig et al. 2021; Zhou et al. 2024). Many of these GCFs can support significant soil degradation in agricultural lands (Sünnemann et al. 2023), with a third of all soils already significantly degraded worldwide (FAO 2023; IPCC 2023). Supporting agricultural sustainability under multiple GCFs is integral to meet the estimated global demand of food and fiber by 2050 (FAO 2023). Despite recent advances, most studies have focused on individual GCFs in isolation, limiting our understanding of how multiple drivers interact to affect crop production and ecosystem functioning (Zhou et al. 2020). Advancing our understanding of how multiple GCFs affect ecosystem multifunctionality is critical for long‐term agriculture sustainability and functioning of agroecosystems.

Soil biodiversity, representing the largest fraction of biodiversity on Earth (Anthony et al. 2023), is critical for sustaining multiple ecosystem functions from plant productivity to organic matter decomposition and pathogen control (Delgado‐Baquerizo et al. 2020). The contribution of soil biodiversity, including bacteria, fungi, protists and invertebrates, in supporting multifunctionality has been demonstrated in natural, agricultural and urban environments from local experiments (Romero et al. 2023) to global scale observational studies (Delgado‐Baquerizo et al. 2016). Furthermore, soil biodiversity is crucial in shaping how multifunctionality responds to climate change (e.g., drought, warming) in natural and agricultural environments (Delgado‐Baquerizo et al. 2017; Trivedi et al. 2019; Yang et al. 2022). Similarly, greater soil biodiversity could buffer the negative impacts of multiple GCFs by supporting higher rates of organic matter decomposition ensuring the cycling and availability of carbon and nutrients belowground (Wang et al. 2025). However, the role of soil biodiversity in sustaining agroecosystem multifunctionality under multiple simultaneous GCFs remains virtually unknown. Advancing knowledge regarding the regulatory contributions of soil biodiversity to the impacts of multiple GCFs is key to supporting agricultural sustainability for the future. Given the prevalent role of soil biodiversity in supporting function, and the previously described capacity of soil microbes to modulate the responses of ecosystem function to individual global change factors, we hypothesized that soil biodiversity may also play a key role in supporting EMF by maintaining ecosystem processes as nutrient cycling, organic matter decomposition, and plant–soil symbiotic interactions under increasing multiple GCFs, since greater microbial diversity is key to maintain processes as organic matter decomposition facilitating the entrance of nutrients under stress and enhance the resistance of ecosystem multifunctionality to global change (Trivedi et al. 2019; Delgado‐Baquerizo et al. 2017).

To test this hypothesis, we conducted a field mesocosm experiment exploring the contribution of soil biodiversity to support EMF under an increasing number of GCFs. Specifically, we conducted a large‐scale potted field experiment manipulating four levels of soil biodiversity through a “dilution‐to‐extinction” approach and exposing mesocosms to an increasing number of up to eight distinct GCFs. We used wheat as our model plant because it is one of the most important staple foods worldwide and plays a critical role in global food systems and socio‐economic wellbeing under global change (Webber et al. 2018). We measured a set of key indicators for multiple ecosystem functions (EMF), including vegetative wheat biomass at flowering (i.e., a surrogate of plant productivity), plant health, organic matter decomposition, and arbuscular mycorrhizal (AM) fungal abundance. We determined averaging EMF and multiple individual functions as in Maestre et al. (2012). Our objective was to assess the contribution of soil biodiversity to supporting EMF under multiple GCFs. Together, our work aims to advance our understanding of the interactions between soil biodiversity loss and multiple simultaneous environmental stressors in agricultural ecosystems.

## 2 Methods

### 2.1 Experimental Design

The experiment was established in 2023 at The Hampa experimental farm, at IRNAS‐CSIC (37.2835° N, 6.0657° W) in Coria del Río, Seville, Spain, with a Mediterranean climate. A total of 400 pots (30 L each) were organized into 100 “plots,” with unique groupings of four pots per plot (Table S1). The experimental area was approximately 200 m^2^. The experimental design was fully factorial, combining eight distinct global change factors and four levels of diversity dilutions and one plant species, wheat ( *Triticum aestivum* ), a crop of global agronomic relevance and sensitive to multiple environmental stressors (Webber et al. 2018). The GCFs were randomly selected to generate three levels of increasing GCF count: three, six, and eight GCFs per pot (see the distribution and replicates in Table S1), following the methodologies of Rillig, Ryo, et al. (2019) and Speißer et al. (2022). We also included pots with zero and one level of stress with four replicates (four replicates per dilution level) to account for control or individual global change factors (Rillig, Ryo, et al. 2019).

### 2.2 Dilution‐To‐Extinction Approach

**FIGURE 1:**
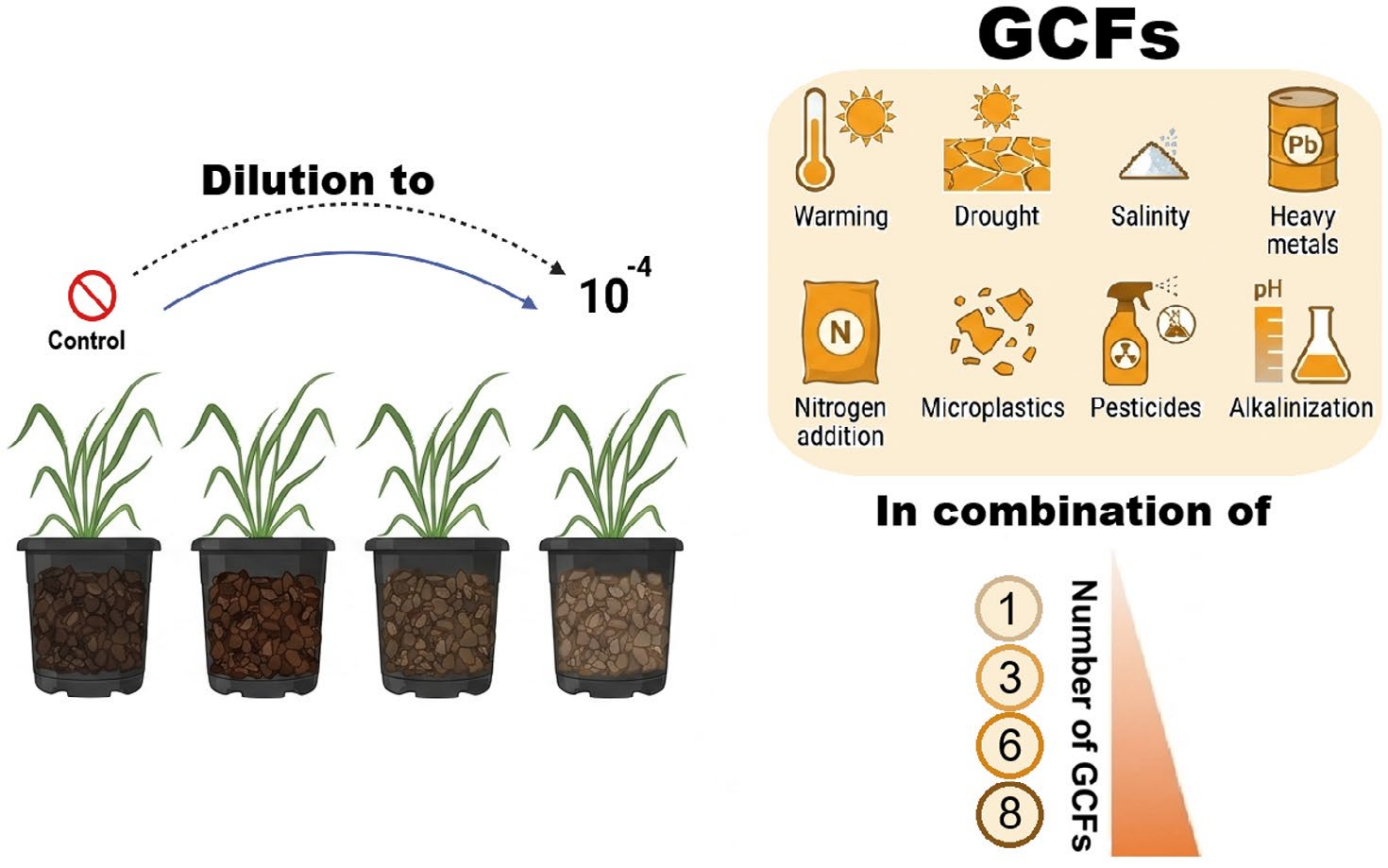
Experimental design. The multi‐factorial experiment consisted of 400 pots (30 L) in total. These were organized into 100 “plots” with unique combinations of four pots in each. A microbial diversity gradient was established using the dilution‐to‐extinction method (control, 10^−x^, 10^−2^, and 10^−4^). Each plot was subjected to individual or randomized combinations of eight global change factors (GCFs; see Table S1 for details), creating a gradient of increasing stress arranged into five treatment levels (0, 1, 3, 6, and 8 GCFs). Figure created with FigureLabs (figurelabs.ai) and assembled in Inkscape.

Four levels of soil biodiversity were established in pots using the “dilution‐to‐extinction” method (Trivedi et al. 2019). Soils were sterilized using an autoclaving approach. A parental inoculum suspension was prepared by mixing 250 g of native farm soil with 5 L of sterilized Milli‐Q water. After vigorous blending and allowing the sediment to settle, serial dilutions (undiluted (0); 1/10 dilution (−x); 1/10^2^ dilution (−2); and 1/10^4^ dilution (−4)) were prepared from the suspension, serving as the microbial inoculum (0.5 L of inoculum for each pot). These inocula were then added to the autoclaved background native farm soils (Figure 1). Each experimental pot (30 L) was filled with ~12 kg of soil. These dilutions were incubated for 6 weeks to allow microbial colonization.

### 2.3 Global Change Factor Treatments

In late November 2023, once the microbial dilutions were incubated, the GCFs combinations were applied, and these treatments were allowed to establish for 2 months. Wheat seeds were sown after this initial phase at the end of January 2024, and daily irrigation of 500 mL of water per pot by drip irrigation was established post‐sowing. We left time between mesocosm establishment and wheat seeding to allow microbial biomass to recover before the beginning of the experiment based on previous experience working with the “dilution‐to‐extinction” approach (Trivedi et al. 2019; Liu et al. 2023; see Figure S4). Our experiment was harvested in the beginning of June 2024 immediately after flowering. All pots were watered during the experiment.

The eight global change factors (GCFs) applied in our experiment were selected due to their differences in their modes of action and their feasibility of implementation under field conditions. Each factor is individually known to affect plants, soils, or their interactions, and their prevalence and intensity are expected to increase in the near future (Hu et al. 2024; Rillig et al. 2021) (See Table S2 for a summary).

Warming: Using open‐top chambers (OTCs) of polycarbonate (1 m × 1 m × 2 mm), we achieved an increase of ~1.97°C above the ambient temperature compared with controls, aiming to simulate expected global warming scenarios (IPCC 2023). Temperature and moisture were continuously recorded using a TMS Standard system. Our OTCs were designed for experiments holding large plants measuring 1 m in height and 1 m in diameter.

Drought: In Southern Europe, drought is one of the most important factors limiting yield production of some of the most important cereals (Webber et al. 2018). Further, climate change has drastically shifted drought regimes globally, with studies highlighting the increasing duration and intensity of pulse droughts across agricultural systems (Yuan et al. 2023; Vicente‐Serrano et al. 2022). We represented a drought episode through a two‐week period with no watering (drip irrigation turned off) imposed mid‐experiment (April 2024), simulating an abrupt drought event without a gradual transition. There was zero rain recorded during this drought period.

Nitrogen addition: N‐addition, even at atmospheric deposition levels, can have profound impacts on agricultural systems and alter nutrient concentrations across watersheds through runoff. Previous studies have shown that N deposition and N addition can lead to significant changes in plant biodiversity and soil biogeochemical equilibrium, increase soil acidification, and force changes in plant physiology (Borer and Stevens 2022; Chen et al. 2023; Liang et al. 2023). A dissolved NPK fertilizer (Universal blue fertilizer NPK (Mg) 12–10–18. BATLLE) solution (300 kg ha−1) was added in a single application to the treated pots to reflect annual accumulation rates, this addition is equivalent to an input of 100 kg N ha−1, comparable to annual atmospheric N deposition in much of Europe (Galloway et al. 2008). To prevent nutrient limitation in control and treated pots, all pots received a basal fertilization of 3 g.

Alkalinization: Alkaline conditions are known to substantially alter soil physicochemical properties and disrupt key microbial processes, such as nitrification and denitrification, affecting ecosystem functioning (Guo et al. 2021). A 1.125 mol/L of dissolved NaOH was added per treated pot to achieve a soil pH of 10. While representing a strong alkaline scenario, this treatment was intended to impose a marked chemical stress capable of producing measurable ecological responses across soil biodiversity.

Heavy Metals: Dissolved copper (ii)‐sulfate—pentahydrate (PanReac AppliChem) was added to soil to a final concentration of 100 mg Cu kg^−1^, simulating a copper contamination hotspot since copper is a common pollutant in Europe and is also used in organic agriculture and viticulture (Ballabio et al. 2018).

Microplastics: Evidence shows that microplastics accumulate in soils globally, and can represent a novel global change stressor (Rillig, de Souza Machado, et al. 2019; Rillig, Ryo, et al. 2019; de Souza Machado et al. 2019). Synthetic rubber ethylene‐propylene‐diene‐monomer (EPDM Granulat) microplastics were used at a concentration of 1% (w/w), The chosen concentration is well within the range of concentrations used in previous studies testing GCFs (Speißer et al. 2022; de Souza Machado et al. 2019).

Salinity: Soil salinization affects 20% of global arable lands, is very relevant in European soils and represents a major human‐induced driver (Daliakopoulos et al. 2016; Arora 2019). Dissolved NaCl was added to our soils to achieve a conductivity of 6 dSm^−^1, considered moderate saline for European soils (Rillig, Ryo, et al. 2019; Speißer et al. 2022).

Pesticide: Imidacloprid, a widely used neonicotinoid in agriculture, was applied at a concentration of 50 ng g^−1^ (PATRON ULTRA Imidacloprid 0.35% 100 g), following the methodology of Rillig, Ryo, et al. (2019) and Wood and Goulson (2017).

A summary of the materials and concentration of each individual GCFs used in our experiment is provided in Table S2.

### 2.4 Ecosystem Multifunctionality

To investigate how soil biodiversity modulates the impact of stressors on ecosystem multifunctionality, we measured 19 different ecosystem indicators at the end of the experiment (5.5 months after sowing): aboveground vegetative wheat biomass at flowering, plant coverage, flowering, growth rate, percentage of chlorotic leaves, total dissolved inorganic phosphorus and nitrogen, soil respiration and glucose‐induced respiration (SIR), organic matter consumption, enzymatic activity for β‐glucosidase (BG), phosphatase (PHOS), N‐acetylglucosaminidase (NAG) and cellobiohydrolase (CB), richness of prokaryotes, fungi, protists, and invertebrates communities, and AM fungal relative abundance. Together, these indicators represent key components of ecosystem functions such as plant productivity, plant health, nutrient cycling, and organic matter decomposition.

To determine aboveground vegetative wheat biomass, wheat plants were harvested, stored in paper bags, dried at 70°C for 72 h to reach a constant mass. Plant productivity was assessed as vegetative wheat biomass (aboveground biomass measured at the flowering stage), a commonly used proxy in global change and biodiversity ecosystem functioning experiments (Garland et al. 2021). While this metric does not capture final grain yield, it provides a standardized estimate of plant growth responses under experimental conditions.

Throughout the experiment, measurements of % germination, plant height, canopy cover, number of ears as flowering, and percentage of chlorotic leaves were taken in the field per pot. The final percentage of chlorotic leaves per pot at harvest was quantified and representative of plant health. Soil organic matter (OM) content was analyzed by the dry combustion method on soil oven‐dried at 105°C (Nelson and Sommers 1996), with OM consumption obtained as the inverse of this variable (−1 × OM content). Total dissolved inorganic nitrogen and phosphorus (hereafter Nitrogen and Phosphorus) were measured by indophenol blue method and malachite green method, respectively (Sims et al. 1995). To measure soil respiration and glucose SIR, we used the MicroResp approach quantifying absorbance at 570 nm after 5 h of incubation at 20°C (Campbell et al. 2003). ~0.5 g of air‐dried soil was placed into each well of a 96‐deepwell microplate and amended with 10 mg glucose and/or water. Soil samples were preincubated for 5 h at 20°C with soil moisture adjusted to 60% of the soil water holding capacity (WHC). The enzyme activities were measured on 1 g of soil using fluorometry for 1.5 h of incubation at 35°C as described by Bell et al. (2013). Soil respiration, Nitrogen, Phosphorus and enzyme activities were conducted using air‐dried soil and results are expressed per gram of air‐dried soil.

Our dataset, including 19 indicators of functions, contained 0.08% missing data affecting five out of 400 mesocosms (1.25%) resulting from plant mortality or the loss of samples during sample collecting. These missing data were restricted to wheat biomass and the percentage of chlorotic leaves (i.e., % of yellowing observed on all leaves). The ecosystem multifunctionality index (EMF) was calculated using the averaging multifunctionality method (Maestre et al. 2012). Each measured attribute was standardized to a 0–1 range (unit‐based standardization), and EMF was computed by averaging all available standardized ecosystem functions at each mesocosm, maximizing data retention. Previous studies have demonstrated that low levels of missing data have little influence on aggregate multifunctionality indices (Soliveres et al. 2016). All metadata results presented in this study are available through Figshare (https://doi.org/10.6084/m9.figshare.32594544).

### 2.5 Soil Biodiversity

Soil biodiversity (microbial richness; number of ASVs) of different soil organisms was determined from rarefied phylotype tables (26,064 and 37,820 sequences for prokaryotes and eukaryotes, respectively), obtained via DNA soil extraction using Powersoil DNA Isolation Kit (QIAGEN. DNeasy PowerSoil Pro Kit Hilden, Germany: QIAGEN; 2023.) and amplicon sequencing targeting the eukaryotic 18S rRNA gene and prokaryotic 16S rRNA gene (Prokaryotes = Bacteria + Archaea) using the primers Euk1391f‐EukBr and 799F‐1193R, respectively. Sequencing was performed using the Illumina Miseq platform. No samples were lost during rarefaction. We followed the approach described by Delgado‐Baquerizo et al. (2018) to define protists as all eukaryotic taxa, separated from fungi, invertebrates (Metazoa) and vascular plants (Streptophyta). On average, prokaryotic communities were dominated by Actinobacteria, Proteobacteria and Bacteroidetes; fungal communities were dominated by Ascomycota, Mucoromycota and Basidiomycota; protist communities were dominated by SAR (Stramenopiles, Alveolates, Rhizaria), Phragmoplastophyta and Amoebozoa; and invertebrate communities were dominated by Nematoda, Arthropoda and Annelida (Figure S3). The relative abundance of AM fungi was identified after passing fungal sequencing data in the FungalTraits database (Põlme et al. 2020). Raw amplicon sequencing data generated in this study have been deposited in the NCBI Sequence Read Archive (SRA) under BioProject accession PRJNA1496365.

### 2.6 Statistical Analysis

We fitted separate linear models to assess the relationship between ecosystem multifunctionality (EMF) and the diversity of each soil organism group (prokaryotes, fungi, protists, and invertebrates), stratifying models by the number of applied global change factors (GCFs). Analyses were performed with all 400 mesocosms, except for wheat biomass (*n* = 397) and % of chlorotic leaves (*n* = 398), which contained missing values. Model performance was evaluated using the coefficient of determination (*R* ^2^) and the significance of regression slopes. To assess the relative importance of GCFs and soil biodiversity in shaping EMF, we used a random forest model (“randomForest” (version 4.6–14) package in the R software) with permutation‐based significance (rfPermute R package). Predictors in these models included the total number of GCFs, each individual GCF, and the diversity of the four groups of soil organisms. Variable importance was quantified using the percentage increase in mean squared error (%IncMSE) upon permuting each predictor, and statistical significance was evaluated with 1000 permutations. Predictors with %IncMSE values significantly greater than zero (*p* < 0.05) were considered influential. To evaluate the robustness of the relationship between the number of GCFs and ecosystem responses independently of the effects associated with nutrient enrichment (N‐addition), we performed ANCOVA analysis and a sensitivity analysis excluding all treatments containing N‐addition, recalculating the number of GCFs accordingly to compare corresponding responses with and w/o enrichment.

We fitted linear models including the interaction between soil biodiversity treatments (dilution levels) and the number of GCFs on EMF. Differences in slopes were further evaluated using analysis of estimated marginal means (emtrends function). Spearman correlations were conducted between each global change factor and the richness of soil microbial groups for both multifunctionality and individual ecosystem functions. Also, permutational multivariate analysis (PERMANOVA) based on Bray–Curtis dissimilarities and nonmetric multidimensional scaling (NMDS) ordination based on Bray–Curtis distance were performed to assess the effect of soil biodiversity and GCF treatments on the community composition of prokaryotic, fungal, protist, and invertebrate communities.

We calculated the predicted EMF for each treatment by summing the individual stressor effects relative to control conditions (zero GCFs) to assess whether the combined effects of multiple GCFs on EMF were additive, synergistic, or antagonistic (Piñeiro et al. 2008; Crain et al. 2008; Rillig, Ryo, et al. 2019).

All analyses were conducted in R (version 4.5.1). For data handling, processing, and visualization, we used the vegan package in R (Oksanen et al. 2022), reshaping (Wickham 2007), tidyverse (Wickham et al. 2019), cowplot (Wilke 2019), qgraph (Epskamp et al. 2012), igraph (Csardi and Nepusz 2006), factoextra (Kassambara and Mundt 2020), phyloseq (McMurdie and Holmes 2013), itsadug (van Rij et al. 2017), rfPermute (Archer 2016), and randomForest (Liaw and Wiener 2002).

## 3 Results

### 3.1 Our Experimental Design Achieved a Soil Biodiversity Gradient

Soil biodiversity manipulation through “dilution‐to‐extinction” treatments generated a gradient of soil species richness across prokaryotes, fungi, invertebrates, and protists. Dilution significantly reduced ASV richness within each group (Figure S1), while having a relatively small impact on community composition (PERMANOVA: prokaryotes *R* ^2^ = 0.096; fungi *R* ^2^ = 0.046; invertebrates *R* ^2^ = 0.025; protists *R* ^2^ = 0.038; all *p* ≤ 0.001), showing that dilution primarily affected taxonomic richness rather than overall community structure (Figures S2 and S3). Moreover, we did not find significant differences in overall soil microbial biomass across mesocosms (Figure S4). Soil community composition remained stable across dilutions, with a differentiation in prokaryotic communities between natural (non‐diluted) soils and within diluted mesocosms (Figure S2).

### 3.2 Soil Biodiversity Supports Ecosystem Multifunctionality Under GCFs

**FIGURE 2:**
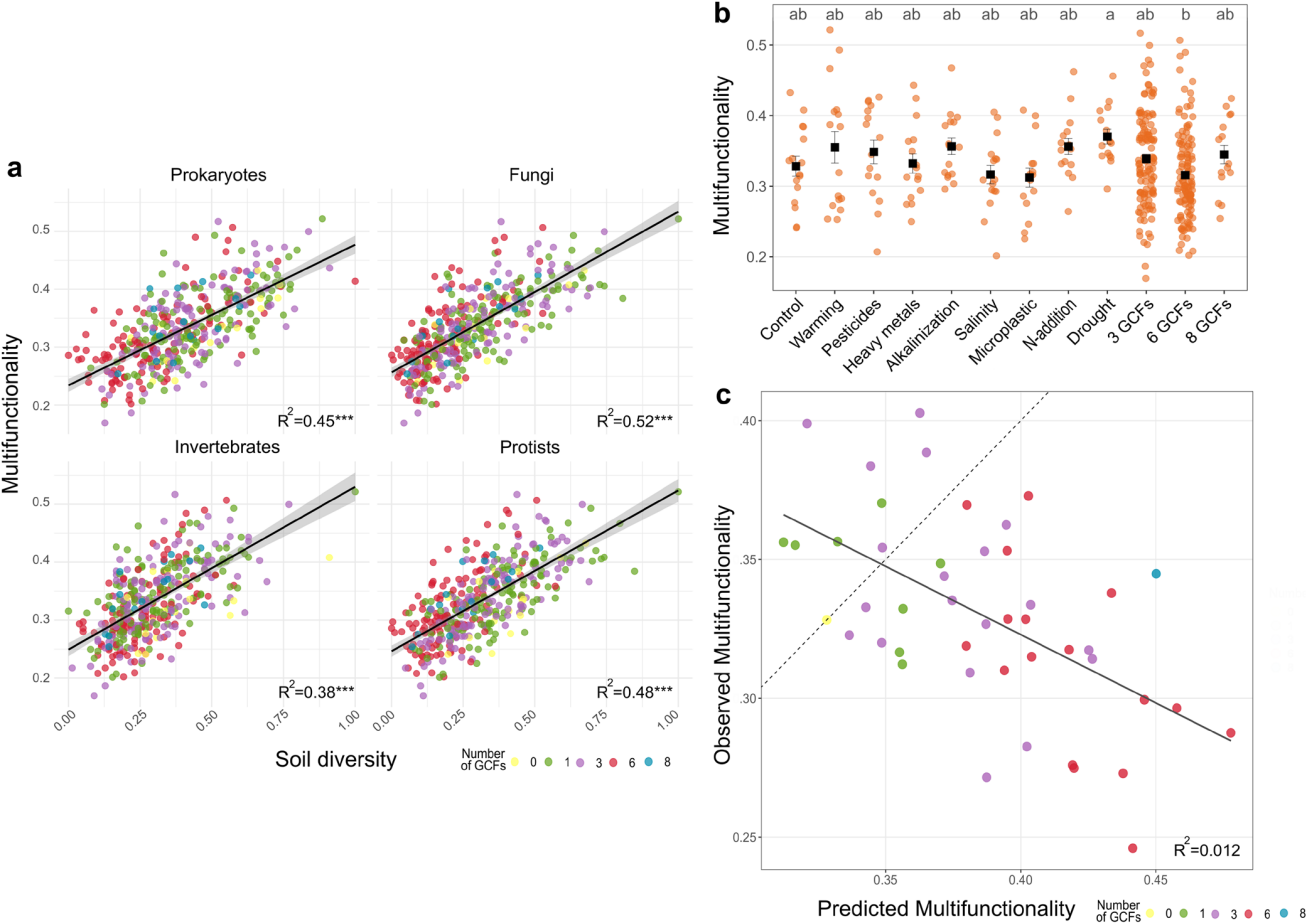
Relationship between ecosystem multifunctionality and soil biodiversity (taxonomic richness) across different soil microbial groups under increasing numbers of global change factors (GCFs). (a) correlations between richness standardized to a 0–1 range, unit‐based standardization of microbial groups (facets) and Multifunctionality (EMF), where the solid black line represents linear regression for all GCF levels. Shaded areas indicate 95% confidence intervals. R^2^ values and significance levels (*** = *p* < 0.001) are shown for each group. (b) Effects of single and multiple (3, 6, and 8 interacting factors) global change factors on EMF (ANOVA: F_11,78.6_ = 3.079, *p* = 0.0017). Individual measurements are in orange and boxplots are in black. Different letters denote significant differences at *p* < 0.05 (c) Observed versus predicted (based on the combination of individual GCF treatments) EMF. Mean EMF values for each treatment are plotted against additive predictions based on single‐stressor effects. Solid gray line represents linear regression for observed and predicted EMF. Dashed line indicates perfect additivity (1:1). R^2^ values and significance levels (*** = *p* < 0.001) are shown. *n* = 400.

**FIGURE 3:**
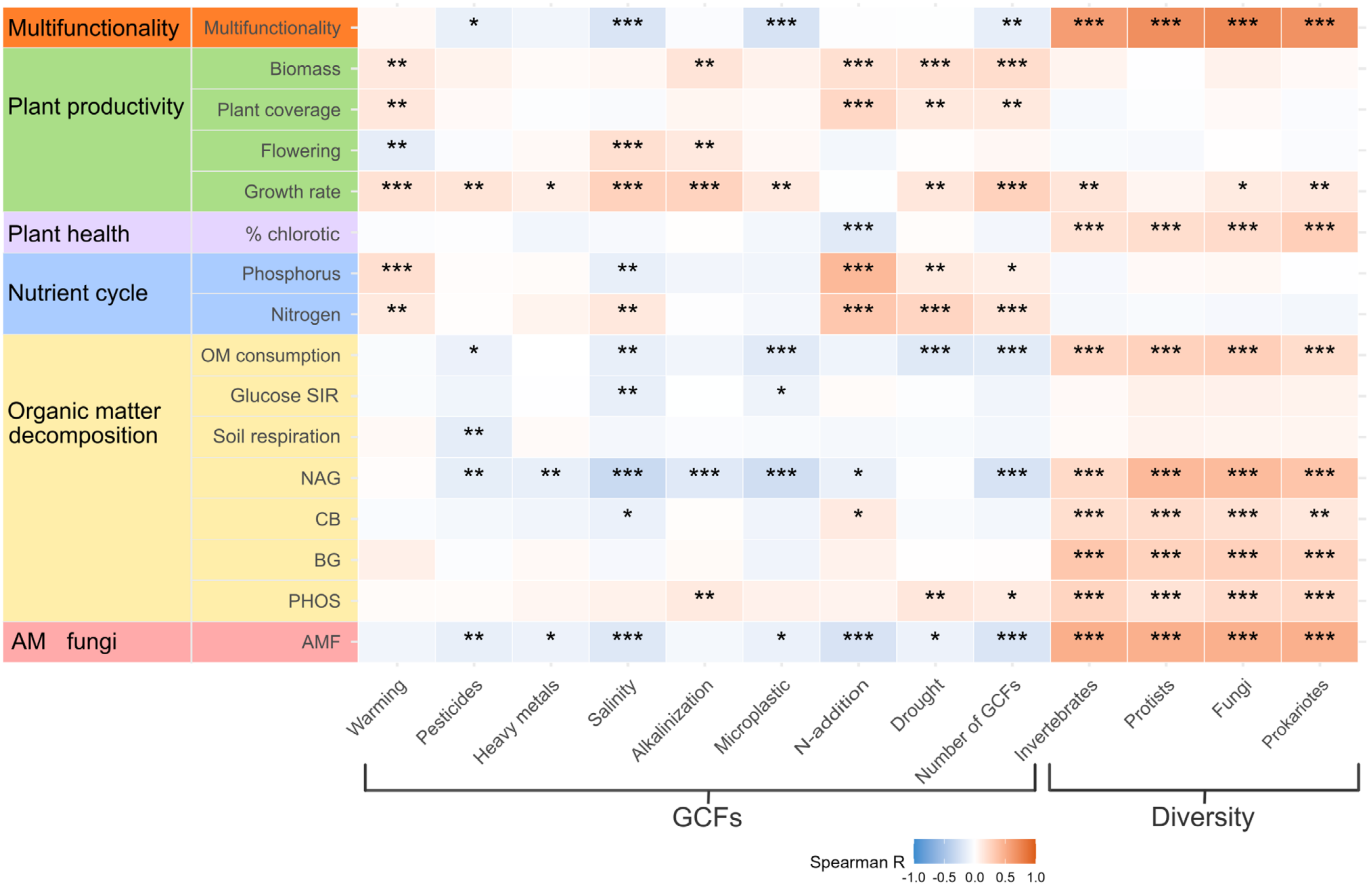
Relationships between global change factors (GCF), soil biodiversity, ecosystem multifunctionality and ecosystem traits. Heatmap showing Spearman correlation coefficients between GCFs, soil biodiversity (richness of prokaryotes, fungi, protists, and invertebrates), averaged ecosystem multifunctionality and individual ecosystem indicators including Wheat biomass, Plant coverage, Flowering, Growth rate, percentage of (%) chlorotic leaves, total dissolved inorganic phosphorus (Phosphorus) and nitrogen (Nitrogen), organic matter consumption (OM consumption), soil respiration and Glucose SIR, enzymatic activity for N‐acetylglucosaminidase (NAG), cellobiohydrolase (CB), β‐glucosidase (BG) and phosphatase (PHOS), AM fungal relative abundance (AMF). “*,” “**,” and “***” represent significant differences between each pair of variables of functions and factors at *p* < 0.05, *p* < 0.01, *p* < 0.001, respectively. *n* = 400 except wheat biomass (*n* = 397) and % of chlorotic (*n* = 398).

**FIGURE 4:**
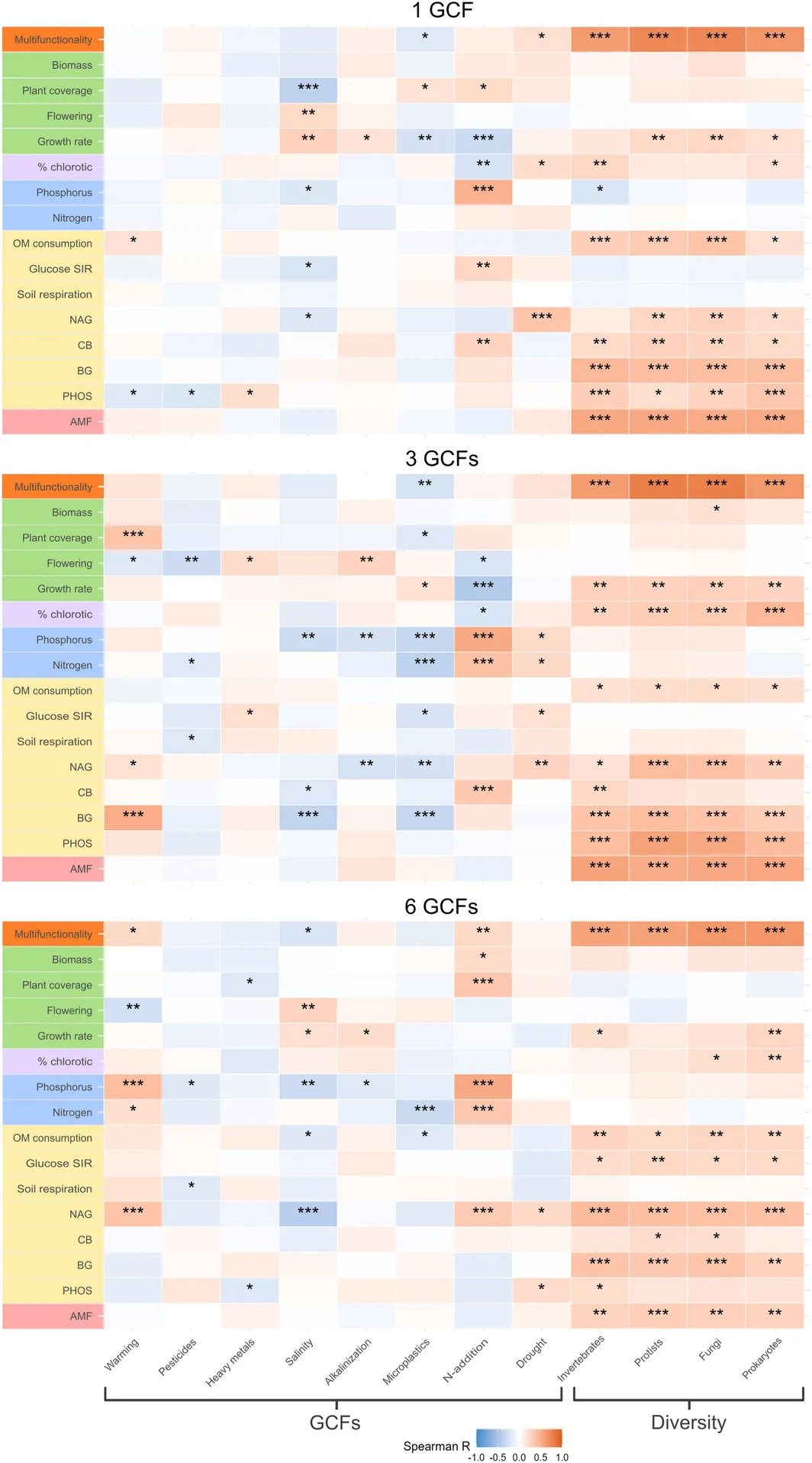
Relationships between global change factor (GCF) levels, soil biodiversity, and ecosystem traits. Heatmap showing Spearman correlation coefficients between GCFs, soil biodiversity (richness of prokaryotes, fungi, protists, and invertebrates), averaged ecosystem multifunctionality and individual ecosystem traits for three levels of global change factors (1, 3, or 6 GCFs). “*,” “**,” and “***” represent significant differences between each pair of variables of functions and factors at *p* < 0.05, *p* < 0.01, *p* < 0.001, respectively. *n* = 400 except wheat biomass (*n* = 397) and % of chlorotic (*n* = 398).

We found significant direct positive linear relationships between soil biodiversity and EMF. Among the different biological groups, fungi showed the strongest association (R^2^ = 0.52) with EMF, while prokaryotes, invertebrates, and protists also displayed consistent positive trends across different levels of GCFs (Figure 2a). Higher diversity of soil organisms correlated positively with indicators such as growth rate, plant health, enzymatic activity, and relative abundance of AM fungi across ecosystem functions, indicating that soil biodiversity supports multiple key ecological processes simultaneously (Figure 3). When exploring responses under increasing numbers of simultaneous GCFs (Figure 4), these relationships remained robust, although the strength of biodiversity and EMF relationships slightly declined under high GCF stress. However, soil biodiversity did not significantly modify the response of EMF to increasing GCF levels. The interaction between soil biodiversity treatment (dilution level) and the number of GCFs was not significant (F_3,392_ = 0.05, *p* = 0.98), indicating that the rate of EMF decline with increasing GCF number did not differ among soil biodiversity levels (Figure S5).

Our results suggest that the positive contribution of soil biodiversity to support function is robust and maintained across contrasting levels of stress. Separating EMF according to GCF stress intensity (Figures S6 and S7) shows that the positive relationship between soil biodiversity and multifunctionality persisted under both low (0–1 GCFs) and high GCF stress (3–6 GCFs). The comparison between observed and additively predicted multifunctionality shows a reduction in multifunctionality that is more pronounced than predicted by individual stressor impacts, suggesting that most of the stressor combinations had synergistic effects on EMF (Figure 2b,c).

### 3.3 Global Change Factor Identity Shapes Ecosystem Function

In general, we found statistically meaningful impacts in both directions for the global change factors (GCFs) on ecosystem functions; we showed that the identity of GCF is as important as the number of stressors in shaping ecosystem functionality, having both positive and negative impacts on different groups of functions and averaged multifunctionality (Figures 3 and 4). When multiple GCFs were present simultaneously, their combined impact led to a general decrease in EMF, organic matter decomposition, and the relative abundance of AM fungi (Figure 3). The negative relationship between the number of GCFs and EMF remained consistent when nitrogen addition was considered as a separate factor and after excluding all treatments involving N addition (Figure S8). Warming generally increased plant productivity and nutrient availability; meanwhile, pesticide application diminished multifunctionality, organic matter decomposition, and AM fungal abundance. Heavy metals negatively affected AM fungal abundance but increased plant growth rate. Salinity had strong negative effects on multifunctionality, decomposition, and abundance of AM fungi, yet promoted some indicators of plant productivity, while soil alkalinization increased productivity but decreased N‐acetylglucosaminidase (NAG) activity. Microplastics decreased multifunctionality, OM decomposition, and abundance of AM fungi but enhanced plant growth rate. N‐addition strongly promoted nutrient cycling and productivity but reduced plant health and AM fungal abundance, while drought also increased some indicators of productivity and nutrient availability but led to lower relative abundance of AM fungi (Figures 3 and 4, S9–S13).

### 3.4 Soil Biodiversity Maintains Ecosystem Functions Under Multiple GCFs

**FIGURE 5:**
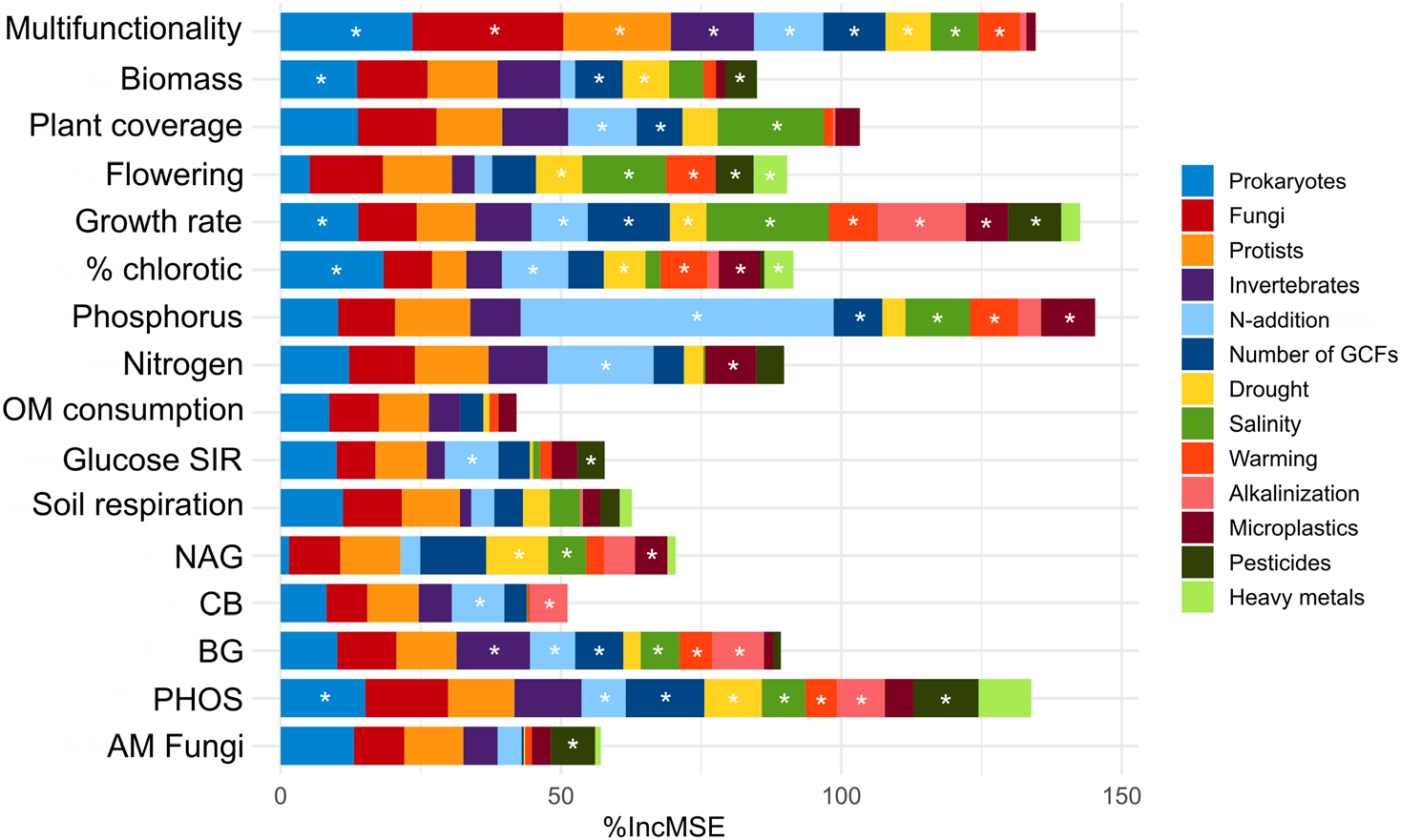
Key predictors influencing ecosystem traits. The importance of predictors, including soil microbial group diversity (richness) and global change factors (GCFs), was determined using random forest machine learning models (%IncMSE). R^2^ values and significance levels (* = *p* < 0.05) are shown for each ecosystem indicator as well as averaged ecosystem multifunctionality. *n* = 400 except biomass (*n* = 397) and % of chlorotic (*n* = 398).

Our results further provided strong evidence that soil biodiversity is essential for supporting multifunctionality regardless of the number and identity of the stressors (Figures 3, 4, 5). We used Random Forest to identify the most important predictors of EMF when considering soil biodiversity, number of GCFs, and identity of the GCF. The most important factor controlling EMF depended on the specific evaluated function; however, soil biodiversity was always a major driver (Figure 5). For example, prokaryotic and fungal diversity were the primary determinants of EMF, with weaker effects from invertebrate and protist richness, as well as the number of GCFs, N‐addition, salinity, and warming (Figure 5). For plant productivity, vegetative wheat biomass was influenced principally by prokaryotic diversity, followed by the number of GCFs, drought, and pesticides. Final plant cover was significantly associated with salinity, over‐fertilization, and the number of GCFs, whereas plant growth rate was determined by multiple factors, including salinity, alkalinization, over‐fertilization, pesticides, warming, microplastics, and prokaryotic richness. Flowering was influenced by salinity, warming, drought, pesticides, and heavy metals. Plant health was mainly driven by prokaryotic diversity, followed by N‐addition and microplastics. N‐addition was the dominant factor affecting nutrient cycling, with minor contributions from the number of GCFs, salinity, and microplastics. For organic matter decomposition, β‐glucosidase (BG) and phosphatase (PHOS) activities were identified as the most influential predictors, while also being influenced by alkalinization, over‐fertilization, number of GCFs, and, in the case of PHOS, by pesticides, drought, heavy metals, and salinity. In contrast, microbial respiration and organic matter consumption were not affected in our experiment. AM fungal relative abundance was primarily explained by pesticide exposure but was also fairly strongly associated with prokaryotic, protist, and total fungal diversity, though nonsignificantly (Figure 5).

## 4 Discussion

Our study provides solid experimental evidence that soil biodiversity is critical to maintain multiple individual functions and averaged multifunctionality in wheat production systems experiencing multiple global change factors. The identity, the number, and the interaction of multiple GCFs impacted multifunctionality. Higher soil biodiversity was especially important in maintaining organic matter decomposition and plant health. Strikingly, the number of stressors and specific GCFs in our experiment generally had a positive effect on wheat growth. Yet, key GCFs such as warming reduced the production of wheat spikes, suggesting that plants may invest more resources in growth and less in reproduction, and therefore, in potential grain production. Taken together, our work suggests that soil biodiversity is a major asset to support crop production and agricultural sustainability under global change through the maintenance of important ecosystem functions and multifunctionality, and therefore, a major priority for ecosystem conservation.

### 4.1 Soil Biodiversity Is Key to Support Multifunctionality

Our results show a significant, positive linear relationship between the richness of soil biodiversity groups (e.g., prokaryotes, fungi, invertebrates, and protists) and multifunctionality, supporting the critical role of soil biota in sustaining ecosystem functions in agricultural systems (Romero et al. 2023). The successful manipulation of the soil biodiversity gradient treatment, where we had a significantly reduced ASV richness while maintaining community composition and microbial biomass (Figures S1, S2, S4), allowed us to test our research question. Overall, microbial richness contributed positively to plant health, organic matter decomposition, and the prevalence of AM fungi (Figure 3). We found that soil biodiversity (i.e., richness of prokaryotes, fungi, protists and invertebrates) is critical in supporting ecosystem multifunctionality across contrasting levels of GCFs (Figure 4) (increasing numbers of GCFs or specific GCFs). Previous work found a positive correlation between soil biodiversity and agroecosystem functioning in maize experiments (Romero et al. 2023), however, the capacity of soil biodiversity to support multifunctionality under contrasting levels of stress remained unknown. Diversity of prokaryotes and fungi were the most important drivers of EMF (%IncMSE of 0.26 and 0.25 respectively) (Figure 5), consistent with their recognized role as regulators of soil health through carbon cycling, nutrient acquisition, and soil aggregation (Chen et al. 2025; Liu et al. 2021). Also, prokaryotic diversity supported plant health and contributed to some indicators of organic matter decomposition and phosphatase activity, indicating that different microbial groups underpin different, yet complementary, ecosystem functions. These results highlight that losses in the functional diversity of key groups such as fungi and prokaryotes can directly compromise the rate of essential soil processes and ecosystem functions (Trivedi et al. 2019).

Remarkably, it was observed that while prokaryotic richness maintained a relatively consistent relationship with multifunctionality irrespective of GCF level, the diversity of fungi, invertebrates, and protists gained increasing importance at an intermediate level of GCFs (Figures 4 and S7). This suggests that while prokaryotic diversity consistently supports multifunctionality regardless of GCFs intensity, the diversity of the other groups can become increasingly important under multiple GCF stress. This could highlight a mechanism of ecosystem resilience, where some taxa provide stable support and others assume compensatory roles under stress (Bardgett and van der Putten 2014; Ma et al. 2022). Recent research consistently indicates that soil microbial communities can modulate, mitigate, or even intensify the effects of global change drivers, depending on the specific identity and some functional characteristics of the organisms involved (Romero et al. 2023; Delgado‐Baquerizo et al. 2020; Trivedi et al. 2019). This modulating capacity likely arises from their functional diversity, where various taxa contribute to complementary ecosystem processes, ensuring the maintenance of essential functions under environmental stress (Wagg et al. 2019).

The absence of strong soil compositional shifts (Figures S1–S5) suggests that the observed soil biodiversity effects are unlikely to be driven by the selection of dominant, rare, or functionally important taxa. Instead, our results are more consistent with an insurance mechanism, where higher microbial diversity increases the likelihood that some taxa sustain ecosystem functioning under multiple GCFs (Delgado‐Baquerizo et al. 2016, 2017, 2020). However, we did not detect a significant buffering effect of ecosystem functioning to multiple stressors, as the decline in multifunctionality with increasing GCFs was similar across all biodiversity dilutions. Taken together, our results show that higher microbial biodiversity enhances overall multifunctioning but does not necessarily sufficiently promote agro‐ecosystem resistance to the increasing stress from multiple GCFs. This implies that multi‐faceted strategies are required to minimize the negative effects of global change on agricultural ecosystems, including international efforts to minimize or reverse further contributions to these individual global change factors.

### 4.2 Global Change Factor Identity Is Critical for Multifunctionality

In general, an increase in the number of GCFs had a negative impact on EMF. However, we also found that global change factor identity was critical in interpreting our results and that the global change factors (GCFs) have heterogeneous and synergistic effects on multiple ecosystem functions (Figures 2 and 3). These results are consistent with previous research indicating that multiple GCFs rarely produce uniform impacts and frequently lead to different trade‐offs among functions (Rillig, Ryo, et al. 2019; Rillig et al. 2021; Wang et al. 2025; Zhou et al. 2020). This is also consistent with experimental evidence showing that multiple anthropogenic pressures can neutralize the positive effects of soil biodiversity on ecosystem functions (Yang et al. 2022). In our study, we found that the N‐addition factor enhanced wheat productivity and belowground nutrient cycling, while simultaneously reducing plant health and AM fungal relative abundance (Figure 3), highlighting the complex nature of GCF impacts on ecosystem functioning (Han et al. 2020; Wang et al. 2025). Similarly, other factors such as salinity, microplastics, and pesticides demonstrated differential effects depending on the specific function, underscoring the nonlinear response of EMF to multiple concurrent GCFs, while the co‐occurrence of multiple GCFs significantly and consistently diminished ecosystem functioning, including averaged EMF, organic matter decomposition, and AM fungal abundance (Figures 3 and 4). Furthermore, the number of individual GCFs that significantly affected EMF increased at the higher GCF level (Figure S7). While the mechanism for this is unknown, it suggests some degree of non‐additivity or feedback among individual GCFs, and as multiple GCFs compound, their impact on EMF becomes apparent (Figure 2b). This high degree of context dependency and the emergence of some unexpected effects, such as the increase of productivity with increasing numbers of GCFs, confirms the limitations of inferring ecosystem responses from single‐factor experiments, highlighting the critical need for global change biology research to fully embrace the complexity and multitude of drivers impacting terrestrial ecosystems (Rillig, Ryo, et al. 2019; Bi et al. 2024).

### 4.3 The Interaction of Multiple Global Change Factors Influenced Ecosystem Functions

The positive correlations we observe from certain GCFs applied in our experiment suggest complex relations between the stressors (Figure 4). For instance, an increasing number of GCFs seem to positively influence wheat growth rate, but did not significantly affect the number of wheat spikes. This is probably associated with stressed wheat shifting resource allocation away from reproduction and toward immediate shoot or root growth under greater stress (higher GCFs). Multi‐stressor treatments could have led to antagonistic chemical interactions, for instance the addition of nutrients (N‐addition) could have mitigated nutrient limitations (e.g., phosphorus) imposed by high pH and precipitation in alkaline soils, thus yielding a mechanism of compensatory growth, nutrient mobilization, or shifts in resource allocation (Du et al. 2023; Zhu et al. 2024). Under moderate salinity and alkalinization conditions, these abiotic stresses can activate defense responses that optimize cellular functions that can prevent toxicity (Msimbira and Smith 2020), and it has been shown that this can enhance mineral‐associated organic carbon accumulation coupled with elevated microbial carbon use efficiency increasing soil organic carbon stability (Zhang et al. 2025). Together this may indirectly lead to improved plant growth, and perhaps the concentrations we applied were too low to elucidate toxicity responses in wheat. The observed increases in productivity under N‐addition and drought suggest complex interactions, possibly involving altered nutrient availability or species‐specific adaptive responses that warrant further investigation (Wang et al. 2025; Zhou et al. 2020). However, these productivity gains often occur alongside reductions in other ecosystem functions, such as organic matter decomposition or shifts in microbial community composition, highlighting trade‐offs inherent in ecosystem multifunctionality under multiple global change factors. Moreover, the consequences on grain production were not determined in this pot experiment, therefore future work needs to quantify these effects on yield to fully address the future of food security.

## 5 Conclusions

Our study demonstrates that soil microbial biodiversity is a key driver of ecosystem multifunctionality in agricultural systems, with prokaryotes and fungi playing distinct, yet often complementary roles across measured functions. The number and identity of GCFs had overall negative impacts on ecosystem multifunctionality and heterogeneous effects on some individual functions. The diversity of fungi, invertebrates, and protists becomes increasingly important to support multifunctionality under the impacts of multiple GCFs, suggesting mechanisms of ecological compensation and resilience. The mechanisms underlying these interactions, including the contributions of functional and trait diversity, still require further investigation to accurately predict and manage multifunctionality under climate change scenarios. However, given the unfortunate reality that global change factors will only become more numerous and more stressful in the near future, our findings underscore the critical need to maintain soil biodiversity in order to buffer, mediate, or amplify ecosystem responses under multiple concurrent global change drivers.

## Author Contributions

**Manuel Delgado‐Baquerizo:** conceptualization, investigation, supervision, funding acquisition, writing – original draft, writing – review and editing, visualization, formal analysis. **Youzhi Feng:** data curation. **Samuel Castejon:** data curation, methodology. **Catalina Garzón‐Ladino:** conceptualization, methodology, software, formal analysis, validation, investigation, data curation, writing – original draft, writing – review and editing. **Guiyao Zhou:** writing – original draft, writing – review and editing. **Daniel Revillini:** investigation, writing – original draft, writing – review and editing. **Yuanyuan Bao:** data curation.

## Funding

This work was supported by Ministerio de Ciencia e Innovación (PID2020‐115813RA‐I00), HORIZON EUROPE Marie Sklodowska‐Curie Actions (MSCA‐PF‐2021‐101064192).

## Conflicts of Interest

The authors declare no conflicts of interest.

## Supporting information


**Figure S1:** Effect of “dilution‐to‐extinction” treatments on taxonomic richness of prokaryotes, fungi, invertebrates and protists. Boxes represent the interquartile range (IQR), horizontal lines indicate medians, and whiskers extend to 1.5 × IQR. Statistical analysis was performed by two‐way ANOVA followed by a Tukey post hoc test (different letters denote statistical significance at *p* < 0.05). *n* = 400.
**Figure S2:** Prokaryotic (a), fungal (b), invertebrate (c) and protist (d) community composition under different dilution treatments and global change factors (GCFs) levels. Nonmetric multidimensional scaling (NMDS) ordination based on Bray–Curtis dissimilarity. Statistical analysis was performed by PERMANOVA. *n* = 400.
**Figure S3:** Relative abundance of the most dominant prokaryotic, fungal, invertebrate and protist phyla across the four microbial dilution levels (0, −x, −2, −4). Phyla with a mean relative abundance lower than 1% across samples were pooled into the category “Others.” *n* = 400.
**Figure S4:** Effect of microbial diversity dilution on glucose‐induced respiration (SIR), a common proxy of microbial biomass. Statistical analysis was performed by two‐way ANOVA and there are no significant differences between dilutions. *n* = 400.
**Figure S5:** Relationship between number of global change factors (GCFs) and ecosystem multifunctionality (EMF) across varying soil biodiversity (i.e., soil microbial dilution) levels. Results of estimated marginal means are provided comparing the slopes for different dilutions. See *n* in Table S1.
**Figure S6:** Relationship between averaged ecosystem multifunctionality and soil biodiversity (standardized averaged diversity of prokaryotes, fungi, protists and invertebrates) under different levels of global change factors stress levels. Stress levels are categorized as “Low” = 0–1 GCFs, and “High” = 3–8 GCFs. The interaction between richness and stress level was significant for invertebrates (t396 = 2.12, *p* = 0.034), whereas this interaction was not significant for prokaryotes (t396 = 0.09, *p* = 0.928), fungi (t396 = 1.79, *p* = 0.074), or protists (t396 = 1.52, *p* = 0.131). *n* = 400.
**Figure S7:** Correlation (Spearman) between global change factors (GCF), soil biodiversity, and Multifunctionality (EMF) across different levels of GCFs. Heatmap showing Spearman correlation coefficients between global change factors (GCFs), soil biodiversity (richness of prokaryotes, fungi, protists, and invertebrates), and ecosystem multifunctionality for three levels of global change factors (1, 3, 6 GCFs). “*,” “**,” and “***” represent significant differences between each pair of variables of functions and factors at *p* < 0.05, *p* < 0.01, *p* < 0.001, respectively. See *n* in Table S1.
**Figure S8:** Standardized effect sizes (±95% CI) from ANCOVA models including number of global change factors (GCFs) and nitrogen addition, and from sensitivity analyses excluding N‐addition treatments (number of GCF w/o N‐addition). Points represent model estimates and horizontal bars indicate confidence intervals. Vertical dashed line denotes zero effect. The ecosystem indicators are Multifunctionality, Biomass, Plant coverage, Flowering, Growth rate, % chlorotic leaves, total dissolved inorganic phosphorus (Phosphorus) and nitrogen (Nitrogen), organic matter consumption (OM consumption), soil respiration and Glucose SIR, enzymatic activity for N‐acetylglucosaminidase (NAG), cellobiohydrolase (CB), β‐glucosidase (BG) and phosphatase (PHOS), AM fungal relative abundance (AMF). *n* = 232.
**Figure S9:** Effects of single and multiple global change factors (combinations of 3, 6, and 8 interacting factors) on individual ecosystem indicators associated with plant productivity. (a) Biomass (ANOVA global: F(11, 79.5) = 6.067,*p* = < 0.0001), (b) Plant coverage (F(11, 80.1) = 3.384, *p* = 0.0007), (c) Flowering (F(11, 80.5) = 3.594, *p* = 0.0004), (d) Growth rate (F(11, 79.5) = 9.175,*p* = < 0.0001). *n* = 16 (Control), 16 (Warming), 16 (Pesticides), 16 (Heavy metals), 16 (Alkalinization), 16 (Salinity), 16 (Microplastics), 16 (N‐addition), 15 (Drought), 118 (3 GCFs), 120 (6 GCFs), 16 (8 GCFs).
**Figure S10:** Effects of single and multiple global change factors (combinations of 3, 6, and 8 interacting factors) on individual ecosystem indicators associated with plant health (i.e., % chlorotic leaves). ANOVA global: F(11, 80.3) = 3.146, *p* = 0.0014. *n* = 16 (Control), 16 (Warming), 16 (Pesticides), 16 (Heavy metals), 16 (Alkalinization), 16 (Salinity), 16 (Microplastics), 16 (N‐addition), 15 (Drought), 118 (3 GCFs), 119 (6 GCFs), 16 (8 GCFs).
**Figure S11:** Effects of single and multiple global change factors (combinations of 3, 6, and 8 interacting factors) on individual ecosystem indicators associated with soil nutrient cycle. (a) soil total dissolved inorganic phosphorus (ANOVA global: F(11, 79.9) = 6.733,*p* = < 0.0001); and (b) nitrogen (F(11, 79.2) = 3.909, *p* = 0.0001). *n* = 16 (Control), 17 (Warming), 16 (Pesticides), 16 (Heavy metals), 16 (Alkalinization), 16 (Salinity), 16 (Microplastics), 16 (N‐addition), 16 (Drought), 119 (3 GCFs), 120 (6 GCFs), 16 (8 GCFs).
**Figure S12:** Effects of single and multiple global change factors (combinations of 3, 6, and 8 interacting factors) on individual ecosystem indicators associated with organic matter decomposition. (a) organic matter consumption (ANOVA global: F(11, 78.7) = 1.974, *p* = 0.0422), (b) Glucose SIR (F(11, 77.2) = 2.100, *p* = 0.0299), (c) soil basal respiration (F(11, 77.0) = 0.549, *p* = 0.864); enzymatic activity for (d) N‐acetylglucosaminidase (F(11, 77.3) = 8.099,*p* = < 0.0001), (e) cellobiohydrolase (ns; zero variance in heavy metals), (f) β‐glucosidase (F(11, 77.5) = 1.458, *p* = 0.165), (g) phosphatase (F(11, 79.1) = 3.222, *p* = 0.0012). *n* = 16 (Control), 17 (Warming), 16 (Pesticides), 16 (Heavy metals), 16 (Alkalinization), 16 (Salinity), 16 (Microplastics), 16 (N‐addition), 16 (Drought), 119 (3 GCFs), 120 (6 GCFs), 16 (8 GCFs). Organic matter consumption = Soil organic matter × −1. Glucose SIR = Glucose‐induced respiration.
**Figure S13:** Effects of single and multiple global change factors (combinations of 3, 6, and 8 interacting factors) on individual ecosystem indicators associated with AM fungal relative abundance. ANOVA global: ns; zero variance in 8 GCFs. *n* = 16 (Control), 17 (Warming), 16 (Pesticides), 16 (Heavy metals), 16 (Alkalinization), 16 (Salinity), 16 (Microplastics), 16 (N‐addition), 16 (Drought), 119 (3 GCFs), 120 (6 GCFs), 16 (8 GCFs).
**Table S1:** GCF combinations used for the different GCF‐number levels. There were four replicates for the lowest (GCF = 0), and the highest (GCF = 8) GCF‐number levels, 30 replicates for the intermediate (3 and 6) and 32 for the individual Level 1 for a total of 100 plots.
**Table S2:** Summary of the materials/equipment used to implement the single GCF treatments, and the intensity or concentration of each GCF.

## Data Availability

Metadata, field and laboratory measurements and biodiversity datasets used to calculate ecosystem multifunctionality (EMF), processed EMF datasets, and the data underlying all statistical analyses reported in this study are publicly available in the Figshare repository: https://doi.org/10.6084/m9.figshare.32594544. Raw amplicon sequencing data have been deposited in the NCBI Sequence Read Archive (SRA) under BioProject accession PRJNA1496365.
