# Supplementary material for: Soil Biodiversity Supports Agroecosystem Services Under Multiple Global Change Factors
Source: Glob Chang Biol. 2026 Aug 20;32(8):e71064. doi: 10.1111/gcb.71064 (PMC13492096; doi:10.1111/gcb.71064)
Supplement: Supplementary file 1 [file GCB-32-e71064-s001.docx]

**Supplementary Materials for**

# **Soil biodiversity supports agroecosystem services under multiple global change factors**

**This pdf file includes:**

Tables S1- S2

Figures S1 – S13

**Table S1.** GCF combinations used for the different GCF-number levels. There were four replicates for the lowest (GCF = 0), and the highest (GCF = 8) GCF-number levels, 30 replicates for the intermediate (3 and 6) and 32 for the individual level 1 for a total of 100 plots.

| **Treatment** | **GCFs-number** | | | | | | | | | | | | |
| --- | --- | --- | --- | --- | --- | --- | --- | --- | --- | --- | --- | --- | --- |
|  | **0** | **1** | | | | | | | | **3** | **6** | **8** | **TOTAL** |
| Warming |  | 4 |  |  |  |  |  |  |  | 13 | 20 | 4 | 41 |
| Drought |  |  | 4 |  |  |  |  |  |  | 8 | 26 | 4 | 42 |
| Alkalinization |  |  |  | 4 |  |  |  |  |  | 9 | 25 | 4 | 42 |
| N-addition |  |  |  |  | 4 |  |  |  |  | 16 | 22 | 4 | 46 |
| Salinity |  |  |  |  |  | 4 |  |  |  | 10 | 23 | 4 | 41 |
| Pesticides |  |  |  |  |  |  | 4 |  |  | 13 | 24 | 4 | 45 |
| Microplastic |  |  |  |  |  |  |  | 4 |  | 10 | 22 | 4 | 40 |
| Heavy metals |  |  |  |  |  |  |  |  | 4 | 11 | 18 | 4 | 37 |
| **n** | 4 | 4 | 4 | 4 | 4 | 4 | 4 | 4 | 4 | 30 | 30 | 4 | **100** |

**Table S2.** Summary of the materials/equipment used to implement the single GCF treatments, and the intensity or concentration of each GCF.

| **Treatment** | **Materials / Equipment** | **Intensity / Concentration per pot** |
| --- | --- | --- |
| Warming | Open‐top chambers (OTC). LEXAN^TM^ 9030 PC colourless 3m x 2m x 2 mm | ≈ 2°C increment over the ambient temperature |
| Drought | - | Two weeks without watering in April 2024 |
| N-addition | Universal blue Fertilizer. NPK (Mg) 12-10-18. BATLLE®, Huerto y jardín. | 300 kg/ha |
| Alkalinization | PanReac AppliChem® NaOH ACS, ISO(solution) | Increased pH to ~ 10 |
| Salinity | PanReac® NaCl ACS, ISO (solution) | Electrical conductivity ~ 6 dS/m |
| Pesticides | PATRON ULTRA®  Imidacloprid 0.35% 100 gr. | Addition of 50 ng/g of soil |
| Microplastic | Ethylene-propylene diene monomer (EPDM) | ~ 2 % (w/w) |
| Heavy metals | PanReac AppliChem® CuSO4.5H2O ACS, ISO | 100 mg Cu/kg of soil |

**Figure S1. Effect of ‘dilution-to-extinction’ treatments on taxonomic richness of prokaryotes, fungi, invertebrates and protists.** Boxes represent the interquartile range (IQR), horizontal lines indicate medians, and whiskers extend to 1.5 × IQR. Statistical analysis was performed by two-way ANOVA followed by a Tukey post hoc test (different letters denote statistical significance at *p* < 0.05). n = 400.

**Figure S2. Prokaryotic (a), fungal (b), invertebrate (c) and protist (d) community composition under different dilution treatments and global change factors (GCFs) levels.** Non-metric multidimensional scaling (NMDS) ordination based on Bray-Curtis dissimilarity. Statistical analysis was performed by PERMANOVA. n = 400.

**Figure S3. Relative abundance of the most dominant prokaryotic, fungal, invertebrate and protist phyla across the four microbial dilution levels (0, –x, –2, –4).** Phyla with a mean relative abundance lower than 1% across samples were pooled into the category ‘Others’. n = 400.

**Figure S4. Effect of microbial diversity dilution on glucose-induced respiration (SIR), a common proxy of microbial biomass.** Statistical analysis was performed by two-way ANOVA and there are no significant differences between dilutions. n = 400.

**Figure S5.** Relationship between number of global change factors (GCFs) and ecosystem multifunctionality (EMF) across varying soil biodiversity (i.e., soil microbial dilution) levels. Results of estimated marginal means are provided comparing the slopes for different dilutions. See n in Table S1.

**Figure S6. Relationship between averaged ecosystem multifunctionality and soil biodiversity (standardized averaged diversity of prokaryotes, fungi, protists and invertebrates) under different levels of global change factors stress levels.** Stress levels are categorized as “Low” = 0-1 GCFs, and “High” = 3-8 GCFs. The interaction between richness and stress level was significant for invertebrates (t₃₉₆ = 2.12, p = 0.034), whereas this interaction was not significant for prokaryotes (t₃₉₆ = 0.09, p = 0.928), fungi (t₃₉₆ = 1.79, p = 0.074), or protists (t₃₉₆ = 1.52, p = 0.131). n = 400.

 **Figure S7.** **Correlation (Spearman) between global change factors (GCF), soil biodiversity, and Multifunctionality (EMF) across different levels of GCFs.** Heatmap showing Spearman correlation coefficients between global change factors (GCFs), soil biodiversity (richness of prokaryotes, fungi, protists, and invertebrates), and ecosystem multifunctionality for three levels of global change factors (1, 3, 6 GCFs). “ * ”, “ ** ”, and “ *** ” represent significant differences between each pair of variables of functions and factors at p < 0.05, p < 0.01, p < 0.001, respectively. See n in Table S1.

**Figure S8. Standardized effect sizes (±95% CI) from ANCOVA models including number of global change factors (GCFs) and nitrogen addition, and from sensitivity analyses excluding N-addition treatments (number of GCF w/o N-addition).** Points represent model estimates and horizontal bars indicate confidence intervals. Vertical dashed line denotes zero effect. The ecosystem indicators are Multifunctionality, Biomass, Plant coverage, Flowering, Growth rate, % chlorotic leaves, total dissolved inorganic phosphorus (Phosphorus) and nitrogen (Nitrogen), organic matter consumption (OM consumption), soil respiration and Glucose SIR, enzymatic activity for N-acetylglucosaminidase (NAG), cellobiohydrolase (CB), β-glucosidase (BG) and phosphatase (PHOS), AM fungal relative abundance (AMF). n = 232.

 **Figure S9. Effects of single and multiple global change factors (combinations of 3, 6, and 8 interacting factors) on individual ecosystem indicators associated with plant productivity**. (**a**) Biomass (ANOVA global: F(11, 79.5) = 6.067, p = <0.0001), (**b**) Plant coverage (F(11, 80.1) = 3.384, p = 0.0007), (**c**) Flowering (F(11, 80.5) = 3.594, p = 0.0004), (**d**) Growth rate (F(11, 79.5) = 9.175, p = <0.0001). n = 16 (Control), 16 (Warming), 16 (Pesticides), 16 (Heavy metals), 16 (Alkalinization), 16 (Salinity), 16 (Microplastics), 16 (N-addition), 15 (Drought), 118 (3 GCFs), 120 (6 GCFs), 16 (8 GCFs).**Figure S10. Effects of single and multiple global change factors (combinations of 3, 6, and 8 interacting factors) on individual ecosystem indicators associated with plant health (i.e., % chlorotic leaves)**. ANOVA global: F(11, 80.3) = 3.146, p = 0.0014. n = 16 (Control), 16 (Warming), 16 (Pesticides), 16 (Heavy metals), 16 (Alkalinization), 16 (Salinity), 16 (Microplastics), 16 (N-addition), 15 (Drought), 118 (3 GCFs), 119 (6 GCFs), 16 (8 GCFs).

**Figure S11. Effects of single and multiple global change factors (combinations of 3, 6, and 8 interacting factors) on individual ecosystem indicators associated with soil nutrient cycle**. (**a**) soil total dissolved inorganic phosphorus (ANOVA global: F(11, 79.9) = 6.733, p = <0.0001); and (**b**) nitrogen (F(11, 79.2) = 3.909, p = 0.0001). n = 16 (Control), 17 (Warming), 16 (Pesticides), 16 (Heavy metals), 16 (Alkalinization), 16 (Salinity), 16 (Microplastics), 16 (N-addition), 16 (Drought), 119 (3 GCFs), 120 (6 GCFs), 16 (8 GCFs).

**Figure S12. Effects of single and multiple global change factors (combinations of 3, 6, and 8 interacting factors) on individual ecosystem indicators associated with organic matter decomposition.** (**a**) organic matter consumption (ANOVA global: F(11, 78.7) = 1.974, p = 0.0422), (**b**) Glucose SIR (F(11, 77.2) = 2.100, p = 0.0299), (**c**) soil basal respiration (F(11, 77.0) = 0.549, p = 0.864); enzymatic activity for (**d**) N-acetylglucosaminidase (F(11, 77.3) = 8.099, p = <0.0001), (**e**) cellobiohydrolase (*ns;* *zero variance in heavy metals*), (**f**) β-glucosidase (F(11, 77.5) = 1.458, p = 0.165), (**g**) phosphatase (F(11, 79.1) = 3.222, p = 0.0012). n = 16 (Control), 17 (Warming), 16 (Pesticides), 16 (Heavy metals), 16 (Alkalinization), 16 (Salinity), 16 (Microplastics), 16 (N-addition), 16 (Drought), 119 (3 GCFs), 120 (6 GCFs), 16 (8 GCFs). Organic matter consumption = Soil organic matter x -1. Glucose SIR = Glucose-induced respiration.

 **Figure S13. Effects of single and multiple global change factors (combinations of 3, 6, and 8 interacting factors) on individual ecosystem indicators associated with AM fungal relative abundance.** ANOVA global: *ns;* *zero variance in 8 GCFs*. n = 16 (Control), 17 (Warming), 16 (Pesticides), 16 (Heavy metals), 16 (Alkalinization), 16 (Salinity), 16 (Microplastics), 16 (N-addition), 16 (Drought), 119 (3 GCFs), 120 (6 GCFs), 16 (8 GCFs).
